# Early assessment of the clinical severity of the SARS-CoV-2 omicron variant in South Africa: a data linkage study

**DOI:** 10.1016/S0140-6736(22)00017-4

**Published:** 2022-01-29

**Authors:** Nicole Wolter, Waasila Jassat, Sibongile Walaza, Richard Welch, Harry Moultrie, Michelle Groome, Daniel Gyamfi Amoako, Josie Everatt, Jinal N Bhiman, Cathrine Scheepers, Naume Tebeila, Nicola Chiwandire, Mignon du Plessis, Nevashan Govender, Arshad Ismail, Allison Glass, Koleka Mlisana, Wendy Stevens, Florette K Treurnicht, Zinhle Makatini, Nei-yuan Hsiao, Raveen Parboosing, Jeannette Wadula, Hannah Hussey, Mary-Ann Davies, Andrew Boulle, Anne von Gottberg, Cheryl Cohen

**Affiliations:** a Centre for Respiratory Diseases and Meningitis, National Institute for Communicable Diseases of the National Health Laboratory Service, Johannesburg, South Africa; b Division of Public Health Surveillance and Response, National Institute for Communicable Diseases of the National Health Laboratory Service, Johannesburg, South Africa; c Centre for Tuberculosis, National Institute for Communicable Diseases of the National Health Laboratory Service, Johannesburg, South Africa; d Centre for HIV and STIs, National Institute for Communicable Diseases of the National Health Laboratory Service, Johannesburg, South Africa; e Sequencing Core Facility, National Institute for Communicable Diseases of the National Health Laboratory Service, Johannesburg, South Africa; f School of Pathology, Faculty of Health Sciences, University of the Witwatersrand, Johannesburg, South Africa; g School of Public Health, Faculty of Health Sciences, University of the Witwatersrand, Johannesburg, South Africa; h SA MRC Antibody Immunity Research Unit, School of Pathology, Faculty of Health Sciences, University of the Witwatersrand, Johannesburg, South Africa; i School of Health Sciences, College of Health Sciences, University of KwaZulu-Natal, Durban, South Africa; j School of Laboratory Medicine and Medical Sciences, University of KwaZulu-Natal, Durban, South Africa; k Department of Virology, University of KwaZulu-Natal, Durban, South Africa; l Lancet Laboratories, Johannesburg, South Africa; m National Health Laboratory Service, Johannesburg, South Africa; n Division of Medical Virology, University of Cape Town, Cape Town, South Africa; o Western Cape Government Health and School of Public Health and Family Medicine, University of Cape Town, Cape Town, South Africa; p Department of Clinical Microbiology and Infectious Diseases, CH Baragwanath Academic Hospital, Johannesburg, South Africa

## Abstract

**Background:**

The SARS-CoV-2 omicron variant of concern was identified in South Africa in November, 2021, and was associated with an increase in COVID-19 cases. We aimed to assess the clinical severity of infections with the omicron variant using S gene target failure (SGTF) on the Thermo Fisher Scientific TaqPath COVID-19 PCR test as a proxy.

**Methods:**

We did data linkages for national, South African COVID-19 case data, SARS-CoV-2 laboratory test data, SARS-CoV-2 genome data, and COVID-19 hospital admissions data. For individuals diagnosed with COVID-19 via TaqPath PCR tests, infections were designated as either SGTF or non-SGTF. The delta variant was identified by genome sequencing. Using multivariable logistic regression models, we assessed disease severity and hospitalisations by comparing individuals with SGTF versus non-SGTF infections diagnosed between Oct 1 and Nov 30, 2021, and we further assessed disease severity by comparing SGTF-infected individuals diagnosed between Oct 1 and Nov 30, 2021, with delta variant-infected individuals diagnosed between April 1 and Nov 9, 2021.

**Findings:**

From Oct 1 (week 39), 2021, to Dec 6 (week 49), 2021, 161 328 cases of COVID-19 were reported in South Africa. 38 282 people were diagnosed via TaqPath PCR tests and 29 721 SGTF infections and 1412 non-SGTF infections were identified. The proportion of SGTF infections increased from two (3·2%) of 63 in week 39 to 21 978 (97·9%) of 22 455 in week 48. After controlling for factors associated with hospitalisation, individuals with SGTF infections had significantly lower odds of admission than did those with non-SGTF infections (256 [2·4%] of 10 547 *vs* 121 [12·8%] of 948; adjusted odds ratio [aOR] 0·2, 95% CI 0·1–0·3). After controlling for factors associated with disease severity, the odds of severe disease were similar between hospitalised individuals with SGTF versus non-SGTF infections (42 [21%] of 204 *vs* 45 [40%] of 113; aOR 0·7, 95% CI 0·3–1·4). Compared with individuals with earlier delta variant infections, SGTF-infected individuals had a significantly lower odds of severe disease (496 [62·5%] of 793 *vs* 57 [23·4%] of 244; aOR 0·3, 95% CI 0·2–0·5), after controlling for factors associated with disease severity.

**Interpretation:**

Our early analyses suggest a significantly reduced odds of hospitalisation among individuals with SGTF versus non-SGTF infections diagnosed during the same time period. SGTF-infected individuals had a significantly reduced odds of severe disease compared with individuals infected earlier with the delta variant. Some of this reduced severity is probably a result of previous immunity.

**Funding:**

The South African Medical Research Council, the South African National Department of Health, US Centers for Disease Control and Prevention, the African Society of Laboratory Medicine, Africa Centers for Disease Control and Prevention, the Bill & Melinda Gates Foundation, the Wellcome Trust, and the Fleming Fund.

## Introduction

Since the first case of SARS-CoV-2 in March, 2020, South Africa has had three epidemic waves, with the beta (B.1.351) and delta (B.1.617.2) variants of concern dominating the second and third waves, respectively. On Nov 24, 2021, the Network for Genomics Surveillance in South Africa (NGS-SA) reported a new variant of SARS-CoV-2, which had been detected from specimens collected on Nov 14, 2021, in South Africa^1^ and originally assigned to the lineage B.1.1.529, to WHO.^2^ On Nov 26, 2021, on the recommendation of the Technical Advisory Group on SARS-CoV-2 Virus Evolution, WHO designated B.1.1.529 as omicron,^3^ the fifth variant of concern. Concomitantly, there was a rapid increase in COVID-19 cases in Gauteng province,^2^ South Africa, from the week beginning Nov 15, 2021, with SARS-CoV-2 testing laboratories reporting an increase in the number of samples with S gene target failure (SGTF) when tested on the TaqPath COVID‑19 PCR test (Thermo Fisher Scientific; Waltham, MA, USA). Subsequently, increases in COVID-19 cases and samples with SGTF were observed in other provinces in South Africa, precipitating a fourth wave of SARS-CoV-2 infections. By Dec 16, 2021, omicron had been detected in 87 countries, with many countries reporting community transmission.

**Research in context**

**Evidence before this study**

On Nov 24, 2021, the Network for Genomics Surveillance in South Africa reported a new variant of SARS-CoV-2 (B.1.1.529), which had been detected from specimens collected on Nov 14, 2021, in South Africa and was later designated by WHO as the omicron variant of concern. The omicron variant of concern has a high number of mutations, some of which are concerning due to predicted immune evasion and increased infectivity. Data on the clinical severity of the omicron variant compared with previous SARS-CoV-2 variants are needed to guide public health planning and response. Using the search terms “SARS-CoV-2”, “Omicron”, and “severity”, we searched PubMed and Google Scholar for studies published in English between Jan 1, 2020, and Dec 22, 2021, assessing how the clinical severity of infection with the omicron variant compares to the severity of infection with other SARS-CoV-2 variants. We identified a single published study from Denmark that comprised 785 participants infected by the omicron variant of SARS-CoV-2, of whom nine were hospitalised, but included an insufficient number of people to robustly evaluate disease severity.

**Added value of this study**

We assessed the clinical severity of infection with the SARS-CoV-2 omicron variant using S gene target failure (SGTF) on the Thermo Fisher Scientific TaqPath COVID-19 PCR test as a proxy, COVID-19 national case and admission data, SARS-CoV-2 laboratory testing data, and SARS-CoV-2 genomic sequence data. We found that individuals with SGTF versus non-SGTF infections had an 80% lower odds of being admitted to hospital, but we were not able to make any firm conclusions on the risk of severe disease among hospitalised individuals, possibly due to the low numbers of individuals included in this analysis. When compared with delta variant infections from the third wave, SGTF infections were associated with a 70% lower odds of severe disease among hospitalised individuals.

**Implications of all the available evidence**

Our early analyses indicate a significantly reduced odds of hospital admission among individuals with SGTF versus non-SGTF infections diagnosed between Oct 1 and Nov 30, 2021, and a significantly reduced odds of severe disease among SGTF-infected individuals compared with individuals infected earlier with the delta variant in South Africa. Immunity (due to previous infection, vaccination, or both) in individuals infected with the omicron variant could, in part, account for this reduced severity. It is unclear as to what extent the omicron variant might have reduced intrinsic virulence, which could have contributed in part to the reduced severity observed in our study. Data from other settings on the severity of the omicron variant will be important to assess the behaviour of the omicron variant in countries with different levels of previous infection and vaccination.

The omicron variant of concern has a high number of mutations, some of which are concerning due to predicted immune evasion and increased infectivity. Although the omicron variant shares a few common mutations with the C.1.2 (a highly mutated lineage previously identified in South Africa),^4^ beta, and delta variants, it also has 22 additional substitutions (including insertions and deletions) not seen in any other variant of concern or variant of interest to date. Among these substitutions, His69_Val70del in the spike protein, previously observed in the alpha variant (B.1.1.7), is known to cause SGTF on the TaqPath COVID‑19 PCR test.

Data on the clinical severity of the omicron variant compared with previous SARS-CoV-2 variants are needed to guide public health planning and response. DATCOV-Gen^5^ is a prospective surveillance network that links real-time SARS-CoV-2 genome data to detailed epidemiological and clinical data on hospitalised cases to allow rapid assessment of the severity and clinical presentation of emerging SARS-CoV-2 variants of concern. We aimed to assess the severity of SARS-CoV-2 infections with the omicron variant in South Africa using SGTF as a proxy for the omicron variant.^6^

## Methods

### Data sources and linkage

In this data linkage study, we linked national, South African, individual-level data from four sources: (1) national COVID-19 case data for the period Jan 1–Dec 6, 2021, reported in real time to the National Institute for Communicable Diseases (NICD) Notifiable Medical Conditions Surveillance System (NMCSS); (2) SARS-CoV-2 laboratory test data (test used and PCR cycle threshold values) for the period Oct 1–Dec 6, 2021, for public sector laboratories (the National Health Laboratory Service reported all test data) and one large private sector laboratory (reported TaqPath PCR tests only); (3) SARS-CoV-2 genome data for the period March 31, 2020–Dec 6, 2021, for clinical specimens sent to the NICD from private and public diagnostic laboratories (predominantly from the Gauteng, North West, Mpumalanga, and Northern Cape provinces) and collected through the pneumonia surveillance programme^7^ in five provinces (Western Cape, KwaZulu-Natal, North West, Gauteng, and Mpumalanga); and (4) DATCOV (between March 31, 2020, and Dec 21, 2021), which is an active surveillance system for COVID-19 hospital admissions, with comprehensive coverage of all hospitals in South Africa.^8^

The case and hospitalisation data we used are from the aforementioned national, comprehensive, population-based datasets including the whole of South Africa. The national COVID-19 case database (NICD NMCSS) is a laboratory-based surveillance programme that receives real-time electronic data on all laboratory-confirmed SARS-CoV-2 cases in South Africa. Reporting of cases is mandated by law and audited. Each case is investigated by public health officials and identified data errors are corrected on the database. The SARS-CoV-2 laboratory test data used in this analysis are national data. The included laboratories (all public sector laboratories and one private sector laboratory) were chosen as these laboratories were able to provide data on the PCR test method and cycle threshold values, and represented more than half of all SARS-CoV-2 tests done in South Africa between Oct 1 and Dec 6, 2021. The DATCOV surveillance programme has been described in detail previously.^8^ Using DATCOV, we have included data on all hospitalised patients with laboratory-confirmed SARS-CoV-2 throughout South Africa. In public sector hospitals, data are abstracted from patient records and directly captured by dedicated data capturers, and, in private hospitals and the Western Cape province public sector, data are transferred electronically in daily bulk extracts from hospital data systems. DATCOV includes patients admitted to hospital for COVID-19 symptoms, patients with acquired nosocomial SARS-CoV-2 infection, and those who have tested positive incidentally when admitted to hospital for other reasons. Case and test data were obtained on Dec 6, 2021, and DATCOV data were obtained on Dec 21, 2021. For hospitalisation and severity analyses, cases were censored to those with a specimen collected before Dec 1, 2021.

We performed individual-level data linkage using unique identifiers, when they existed; otherwise, we used a combination of name, surname (family name), and date of birth. Following data linkage, a combination of automated and manual checks were used to ensure accuracy. Ethical approval was obtained from the Human Research Ethics Committee (Medical) of the University of the Witwatersrand for the collection of COVID-19 case and test data as part of essential communicable disease surveillance (M210752), and for the DATCOV surveillance programme (M2010108).

### Definitions

Infections were classified as SGTF (as a proxy for the omicron variant) when an individual tested positive on the TaqPath COVID-19 PCR test with a non-detectable S gene target and a cycle threshold of 30 or less for either the ORF1ab or nucleocapsid gene targets.^9^ We restricted to a cycle threshold of 30 or less to avoid incorrectly classifying infections for which the S gene was not detected (because of low viral load) as SGTF (ie, high cycle threshold values). Infections were classified as non-SGTF when an individual tested positive on the TaqPath COVID-19 PCR test with a cycle threshold of 30 or less for either the ORF1ab or nucleocapsid gene targets and had detectable S gene target. The delta variant was identified by genome sequencing.

An individual was classified as having been admitted to hospital if they linked to a case on the DATCOV database with an admission date from 7 days before to 21 days following the date of specimen collection. We included admissions with a test date before admission because clinicians might not suspect SARS-CoV-2 infection at the time of admission, initial tests might be negative, or infection might be nosocomially acquired. Severe disease was defined as a hospitalised patient meeting at least one of the following criteria: admitted to an intensive care unit (ICU); received any level of oxygen treatment; was ventilated; received extracorporeal membrane oxygenation; had acute respiratory distress syndrome; or had died. Comorbidity was defined as the presence of at least one of the following conditions: hypertension, diabetes, chronic cardiac disease, chronic kidney disease, asthma, chronic obstructive pulmonary disease, malignancy, HIV, and active or previous tuberculosis. Data on obesity and pregnancy were not included as these data were not systematically collected. Reinfection was defined by an individual having at least one positive COVID-19 test (either PCR-based or antigen-based) more than 90 days before the current positive COVID-19 test. Vaccination was defined as having at least one dose of a SARS-CoV-2 vaccine (Ad.26.COV2.S [Johnson & Johnson] or BNT162b2 [Pfizer-BioNTech]).

### Statistical analysis

The mean cycle threshold value (a proxy for viral load) for all public sector positive COVID-19 PCR tests (any PCR test) during the early wave of omicron (weeks 46–48; Nov 14–Dec 4, 2021) was compared with that of the early wave of delta (weeks 18–20; May 2–22, 2021) using the Student's *t* test. Early wave periods were defined from the week before the country crossed a weekly incidence of 30 cases per 100 000 people to 2 weeks later.^10^ When the PCR test included one or more gene target, the target with the lowest cycle threshold value was used for the analysis.

We described the epidemiological characteristics of SGTF infections by comparing SGTF-infected individuals with non-SGTF-infected individuals diagnosed between Oct 1 and Nov 30, 2021, hospitalised SGTF-infected individuals with hospitalised non-SGTF-infected individuals diagnosed between Oct 1 and Nov 30, 2021, and hospitalised SGTF-infected individuals diagnosed between Oct 1 and Nov 30, 2021, with hospitalised delta variant-infected individuals diagnosed between April 1 and Nov 9, 2021, using multivariable logistic regression analysis.

The severity of infections with the SARS-CoV-2 omicron variant was assessed in two ways: (1) by comparing individuals with SGTF versus non-SGTF infections diagnosed between Oct 1 and Nov 30, 2021, and (2) by comparing individuals with delta variant infections diagnosed between April 1 and Nov 9, 2021, with SGTF-infected individuals who were diagnosed between Oct 1 and Nov 30, 2021. Hospitalisation and severity data were obtained for cases on Dec 21, 2021, to allow for at least 3 weeks of follow-up for admission to hospital and in-hospital outcomes. Among people with COVID-19 in the DATCOV database hospitalised between March 5, 2020, and Dec 18, 2021, the median time from admission to an in-hospital outcome was 6 days (IQR 3–11 days; n=414 149) overall, 5 days (2–11; n=94 938) for individuals who died, and 6 days (3–10; n=304 845) for individuals who were discharged alive. Therefore, we were not likely to miss many outcomes with a mininum follow-up of 3 weeks. Severity analyses were restricted to admissions that had already accumulated outcomes and all patients still in hospital were excluded because, in the DATCOV programme, detailed information on hospitalisation in public sector hospitals (eg, oxygen treatment or ICU admission) is populated at the time of discharge or death.

Two models were generated to assess hospitalisation and severe disease among hospitalised individuals as outcome variables. Multivariable logistic regression was done to evaluate the association of SGTF infection versus non-SGTF infection with hospitalisation among people with COVID-19 diagnosed between Oct 1 and Nov 30, 2021. We controlled for factors known to be associated with hospitalisation (ie, age, sex, province, and health-care sector [private *vs* public]) and adjusted for known previous SARS-CoV-2 infection. Data on vaccination status and comorbidities were only available for hospitalised individuals; therefore, we could not control for these variables in this analysis. Multivariable logistic regression was done to evaluate the association of SGTF infection versus non-SGTF infection with disease severity among hospitalised individuals diagnosed with COVID-19 between Oct 1 and Nov 30, 2021, restricting to individuals with a known in-hospital outcome on Dec 21, 2021 (excluding those still in hospital). We controlled for factors known to be associated with disease severity (ie, age, the presence of comorbidity, sex, province, and health-care sector) and adjusted for the number of days between the date of specimen collection and the date of hospital admission, known previous SARS-CoV-2 infection, and SARS-CoV-2 vaccination status.

We compared delta variant infections diagnosed between April 1 and Nov 9, 2021, with SGTF infections diagnosed between Oct 1 and Nov 30, 2021. Multivariable logistic regression was done to evaluate the association of SGTF infections versus delta variant infections with disease severity among hospitalised individuals, restricting to individuals with a known in-hospital outcome on Dec 21, 2021 (excluding those still in hospital). We controlled for factors associated with disease severity (ie, age, the presence of comorbidity, sex, province, and health-care sector) and adjusted for the number of days between specimen collection and hospital admission, known previous SARS-CoV-2 infection, and SARS-CoV-2 vaccination status. We did not compare the risk of hospital admission between individuals with delta variant infections and those with SGTF infections because sequenced samples for the delta variant were not population-based and represented only a small subset of delta variant infections that were sent to the NICD for sequencing.

Categorical variables were summarised by use of frequency distributions and compared by use of Pearson's χ^2^ test. Pairwise interactions were assessed by the inclusion of product terms for all variables remaining in the final multivariable additive models. Analyses were done by use of Stata, version 14.1. p values less than 0·05 were considered statistically significant.

### Role of the funding source

The funders of the study had no role in study design, data collection, data analysis, data interpretation, or writing of the report.

## Results

**Figure 1:**
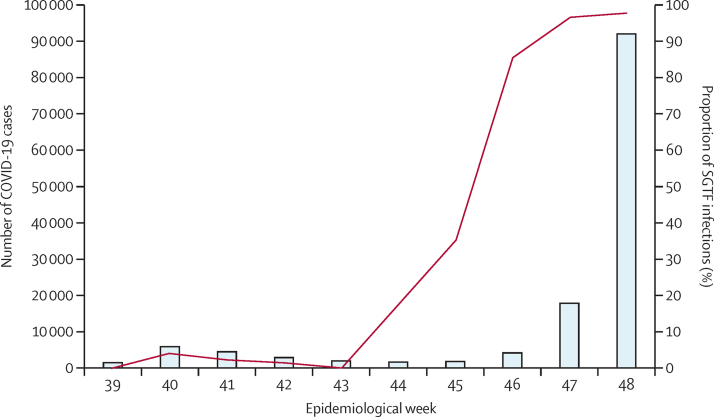
Number of COVID-19 cases detected and proportion of SGTF infections in South Africa by week Diagnosis for the proportion of SGTF infections was by the TaqPath PCR assay. Week 39 represents Oct 1, 2021, and week 48 represents Dec 4, 2021. DATCOV-Gen linked real-time SARS-CoV-2 test and genome data to detailed epidemiological and clinical data on hospitalised cases. SGTF=S gene target failure.

From Oct 1 (week 39), 2021, to Dec 6 (week 49), 2021, 161 328 cases of COVID-19 were reported nationally, of which 104 529 (64·8%) were from laboratories included in our analysis (appendix pp 6–7). Of these 104 529, 38 282 (36·6%) were diagnosed via the TaqPath COVID-19 PCR test. Among the cases diagnosed by TaqPath PCR tests, 31 133 (81·3%) had a cycle threshold value of 30 or less for at least one gene target, of which 29 721 (95·5%) were SGTF infections and 1412 (4·5%) were non-SGTF infections. All SGTF samples with genome data, as of Dec 14, 2021, were confirmed as omicron (n=30). The proportion of SGTF infections increased from two (3·2%) of 63 in week 39 to 21 978 (97·9%) of 22 455 in week 48 of 2021 (figure 1). The proportion of SGTF infections started to increase first in Gauteng province in week 43 (the week starting Oct 24, 2021; appendix p 8). Sharp increases in the proportions of SGTF infections were subsequently detected in all other provinces, and, by week 47, most SARS-CoV-2 infections in all provinces were SGTF infections (appendix p 8). The mean cycle threshold value for all positive PCR tests in the public sector was lower during the early wave of omicron (23·95 [SD 6·06]; n=16 542) than during the early wave of delta variant infection (26·98 [6·52]; n=10 022; p<0·001).

We compared the characteristics of all individuals with SGTF infections diagnosed between Oct 1 and Nov 30, 2021, with those of individuals diagnosed with non-SGTF infections during the same period. In multivariable analyses, in addition to the proportions of SGTF versus non-SGTF infections varying significantly by geographical province, individuals with SGTF infections were more likely to be aged 5–59 years (*vs* ≥60 years) and be diagnosed by the private sector (*vs* the public sector) and were less likely to be admitted to hospital than were individuals with non-SGTF infections (table 1). Similar results were found when comparing characteristics between hospitalised SGTF-infected individuals and hospitalised non-SGTF-infected individuals diagnosed between Oct 1 and Nov 30, 2021 (appendix pp 1–3) and when comparing hospitalised SGTF-infected individuals diagnosed between Oct 1 and Nov 30, 2021, with hospitalised delta variant-infected individuals diagnosed between April 1 and Nov 9, 2021 (appendix pp 3–5).

**Table 1:** Multivariable logistic regression analysis evaluating factors associated with SGTF versus non-SGTF SARS-CoV-2 infection, South Africa, Oct 1–Nov 30, 2021* (n=11 255)

	**Non-SGTF**†	**SGTF**†	**Odds ratio (95% CI)**	**Adjusted odds ratio (95% CI)**	**p value**
**Age group**					
<5 years	21/948 (2·2%)	135/10 547 (1·3%)	1·5 (0·9–2·4)	1·0 (0·6–1·9)	0·88
5–12 years	41/948 (4·3%)	581/10 547 (5·5%)	3·2 (2·3–4·7)	1·6 (1·0–2·5)	0·033
13–18 years	59/948 (6·2%)	610/10 547 (5·8%)	2·4 (1·7–3·3)	1·8 (1·2–2·6)	0·004
19–24 years	88/948 (9·3%)	1027/10 547 (9·7%)	2·7 (2·0–3·6)	2·6 (1·9–3·7)	<0·001
25–39 years	305/948 (32·2%)	4664/10 547 (44·2%)	3·5 (2·8–4·3)	2·8 (2·2–3·7)	<0·001
40–59 years	283/948 (29·9%)	2873/10 547 (27·2%)	2·3 (1·9–2·9)	1·7 (1·3–2·3)	<0·001
≥60 years	151/948 (15·9%)	657/10 547 (6·2%)	1 (ref)	1 (ref)	..
**Sex**					
Male	417/933 (44·7%)	4612/10 471 (44·0%)	1 (ref)	1 (ref)	..
Female	516/933 (55·3%)	5859/10 471 (56·0%)	1·0 (0·9–1·2)	1·1 (0·9–1·3)	0·27
**Province**					
Eastern Cape	0	9/10 435 (0·1%)	..	..	..
Free State	67/918 (7·3%)	55/10 435 (0·5%)	0·6 (0·4–0·9)	1·1 (0·7–1·7)	0·71
Gauteng	424/918 (46·2%)	9083/10 435 (87·0%)	15·6 (12·2–20·0)	12·1 (9·2–16·0)	<0·001
KwaZulu-Natal	197/918 (21·5%)	337/10 435 (3·2%)	1·2 (0·9–1·7)	1·0 (0·7–1·3)	0·81
Limpopo	12/918 (1·3%)	245/10 435 (2·3%)	14·9 (8·0–27·8)	5·6 (2·9–10·7)	<0·001
Mpumalanga	10/918 (1·1%)	231/10 435 (2·2%)	16·9 (8·6–33·1)	6·0 (3·0–12·0)	<0·001
North West	14/918 (1·5%)	260/10 435 (2·5%)	13·6 (7·6–24·3)	6·6 (3·5–12·5)	<0·001
Northern Cape	67/918 (7·3%)	41/10 435 (0·4%)	0·4 (0·3–0·7)	0·8 (0·5–1·3)	0·41
Western Cape	127/918 (13·8%)	174/10 435 (1·7%)	1 (ref)	1 (ref)	..
**Hospital admission**					
No	827/948 (87·2%)	10 286/10 547 (97·5%)	1 (ref)	1 (ref)	..
Yes	121/948 (12·8%)	261/10 547 (2·5%)	0·2 (0·1–0·2)	0·2 (0·1–0·3)	<0·001
**Health-care sector**					
Public	635/948 (67·0%)	2391/10 547 (22·7%)	1 (ref)	1 (ref)	..
Private	313/948 (33·0%)	8156/10 547 (77·3%)	6·9 (6·0–8·0)	5·4 (4·5–6·4)	<0·001
**Reinfection**‡					
No	905/948 (95·5%)	9447/10 547 (89·6%)	1 (ref)	1 (ref)	..
Yes	43/948 (4·5%)	1100/10 547 (10·4%)	2·5 (1·8–3·3)	1·3 (0·9–1·9)	0·18

Data are n/N (%), unless otherwise specified. SGTF=S gene target failure.

* Patients with COVID-19 followed up for hospital admission until Dec 21, 2021.

† SGTF was a proxy for the omicron variant (B.1.1.529).

‡ Reinfection was defined by an individual having at least one positive SARS-CoV-2 test more than 90 days before the current positive SARS-CoV-2 test.

Of the 10 547 individuals with SGTF infections diagnosed between Oct 1 and Nov 30, 2021, 256 (2·4%) were admitted to hospital, compared with 121 (12·8%) of 948 individuals with non-SGTF infections (p<0·001; table 2). In multivariable analyses, after controlling for factors associated with hospitalisation, individuals with SGTF infections had significantly lower odds of being admitted to hospital than did individuals with non-SGTF infections (adjusted odds ratio [aOR] 0·2, 95% CI 0·1–0·3; table 2). In addition to variation by geographical province, hospital admission was significantly associated with young age (<5 years; aOR 9·3) and older age (≥60 years; aOR 3·1) compared with individuals aged 19–24 years, and female sex (aOR 1·3) versus male sex (table 2). Individuals diagnosed by the private versus the public health-care sector were less likely to be admitted to hospital (aOR 0·8; table 2).

**Table 2:** Multivariable logistic regression analysis evaluating the association between SGTF versus non-SGTF SARS-CoV-2 infection and hospitalisation, South Africa, Oct 1–Nov 30, 2021* (n=11 255)

	**Hospital admission**†	**Adjusted odds ratio (95% CI)**	**p value**
**SARS-CoV-2 variant (n=11 495)**			
SGTF	256/10 547 (2·4%)	0·2 (0·1–0·3)	<0·001
Non-SGTF	121/948 (12·8%)	1 (ref)	..
**Age group (n=11 495)**			
<5 years	28/156 (17·9%)	9·3 (5·2–16·8)	<0·001
5–12 years	10/622 (1·6%)	0·8 (0·4–1·7)	0·52
13–18 years	13/669 (1·9%)	0·9 (0·4–1·7)	0·68
19–24 years	25/1115 (2·2%)	1 (ref)	..
25–39 years	161/4969 (3·2%)	1·5 (1·0–2·3)	0·069
40–59 years	77/3156 (2·4%)	1·0 (0·6–1·6)	0·94
≥60 years	68/808 (8·4%)	3·1 (1·9–5·0)	<0·001
**Sex (n=11 404)**			
Male	144/5029 (2·9%)	1 (ref)	..
Female	235/6375 (3·7%)	1·3 (1·1–1·6)	0·016
**Province (n=11 353)**			
Eastern Cape	0/9	..	..
Free State	9/122 (7·4%)	1·4 (0·6–3·6)	0·43
Gauteng	285/9507 (3·0%)	2·0 (1·0–3·7)	0·037
KwaZulu-Natal	40/534 (7·5%)	2·4 (1·2–4·7)	0·013
Limpopo	6/257 (2·3%)	1·7 (0·6–4·8)	0·33
Mpumalanga	4/241 (1·7%)	1·3 (0·4–4·1)	0·71
North West	11/274 (4·0%)	2·9 (1·2–6·9)	0·019
Northern Cape	4/108 (3·7%)	0·8 (0·2–2·4)	0·64
Western Cape	12/301 (4·0%)	1 (ref)	..
**Health-care sector (n=11 495)**			
Public	159/3026 (5·3%)	1 (ref)	..
Private	223/8469 (2·6%)	0·8 (0·6–1·0)	0·024
**Reinfection**‡**(n=11 495)**			
No	351/10 352 (3·4%)	1 (ref)	..
Yes	31/1143 (2·7%)	1·1 (0·7–1·6)	0·66

Data are n/N (%), unless otherwise specified. SGTF=S gene target failure.

* Patients with COVID-19 followed up for hospital admission until Dec 21, 2021.

† Admission to hospital from 7 days before to 21 days following diagnosis (date of specimen collection).

‡ Reinfection was defined by an individual having at least one positive SARS-CoV-2 test more than 90 days before the current positive SARS-CoV-2 test.

Among the 382 hospitalised individuals diagnosed with COVID-19 between Oct 1 and Nov 30, 2021, 317 (83%) had accumulated an in-hospital outcome by Dec 21, 2021. After controlling for factors associated with severe disease, the point estimate for the odds of severe disease in individuals with SGTF versus non-SGTF infections was less than 1, but the 95% CI was wide (aOR 0·7, 95% CI 0·3–1·4; table 3). The odds of severe disease varied geographically and were significantly higher among individuals aged 60 years or older (aOR 11·5) than among individuals aged 19–24 years (table 3).

**Table 3:** Multivariable logistic regression analysis evaluating the association between SGTF versus non-SGTF SARS-CoV-2 infection and severe disease among hospitalised individuals with a known outcome, South Africa, Oct 1–Nov 30, 2021* (n=290)

	**Severe disease**†	**Adjusted odds ratio (95% CI)**	**p value**
**SARS-CoV-2 variant (n=317)**			
SGTF	42/204 (21%)	0·7 (0·3–1·4)	0·30
Non-SGTF	45/113 (40%)	1 (ref)	..
**Age group (n=317)**			
<5 years	2/27 (7%)	0·6 (0·1–4·0)	0·58
5–12 years	1/8 (13%)	0·5 (0·0–6·7)	0·63
13–18 years	0/8	..	..
19–24 years	4/23 (17%)	1 (ref)	..
25–39 years	19/136 (14%)	0·6 (0·2–2·2)	0·43
40–59 years	23/59 (39%)	3·3 (0·8–12·9)	0·094
≥60 years	38/56 (68%)	11·5 (2·8–47·0)	0·001
**Sex (n=315)**			
Male	37/120 (31%)	1 (ref)	..
Female	48/195 (25%)	0·7 (0·3–1·3)	0·24
**Province (n=308)**			
Eastern Cape	0/0	..	..
Free State	3/8 (38%)	8·6 (0·4–167·0)	0·16
Gauteng	56/230 (24%)	18·8 (2·0–176·3)	0·010
KwaZulu-Natal	15/38 (39%)	18·0 (1·7–190·5)	0·016
Limpopo	0/5	..	..
Mpumalanga	2/4 (50%)	33·9 (1·2–946·0)	0·038
North West	3/7 (43%)	44·9 (2·4–837·5)	0·011
Northern Cape	3/4 (75%)	113·6 (3·9–3327·6)	0·006
Western Cape	2/12 (17%)	1 (ref)	..
**Comorbidity**‡**(n=317)**			
Absent	56/255 (22%)	1 (ref)	..
Present	31/62 (50%)	2·2 (1·0–4·8)	0·060
**Health-care sector (n=317)**			
Public	43/130 (33%)	1 (ref)	..
Private	44/187 (24%)	0·9 (0·4–1·9)	0·72
**Days between diagnosis and admission (n=317)**			
1–7 days before diagnosis	8/38 (21%)	1 (ref)	..
0–6 days after diagnosis	75/275 (27%)	3·0 (1·0–9·2)	0·059
7–21 days after diagnosis	4/4 (100%)	..	..
**Reinfection**§**(n=317)**			
No	80/290 (28%)	1 (ref)	..
Yes	7/27 (26%)	2·5 (0·8–8·0)	0·11
**SARS-CoV-2 vaccination**¶**(n=317)**			
No	13/42 (31%)	1 (ref)	..
Yes	4/9 (44%)	0·6 (0·1–4·1)	0·60
Unknown	70/266 (26%)	0·3 (0·1–1·0)	0·055

Data are n/N (%), unless otherwise specified. SGTF=S gene target failure.

* Patients with COVID-19 followed up for in-hospital outcome until Dec 21, 2021.

† Severe disease was defined as a hospitalised patient meeting at least one of the following criteria: admitted to an intensive care unit; received oxygen treatment; was ventilated; received extracorporeal membrane oxygenation; had acute respiratory distress syndrome; or had died.

‡ Comorbidity was defined as the presence of at least one of the following conditions: hypertension, diabetes, chronic cardiac disease, chronic kidney disease, asthma, chronic obstructive pulmonary disease, malignancy, HIV, and active or previous tuberculosis.

§ Reinfection was defined by an individual having at least one positive SARS-CoV-2 test more than 90 days before the current positive SARS-CoV-2 test.

¶ Vaccination was defined as having at least one dose of a SARS-CoV-2 vaccine (Ad.26.COV2.S [Johnson & Johnson] or BNT162b2 [Pfizer–BioNTech]).

**Figure 2:**
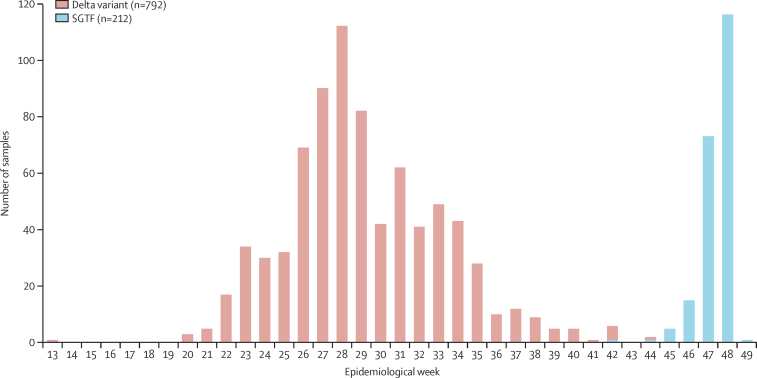
Number of SARS-CoV-2 delta variant and SGTF samples among hospitalised patients with COVID-19 and a known outcome in South Africa by epidemiological week and variant type Week 13 represents April 1, 2021, and week 49 represents Dec 6, 2021. DATCOV-Gen linked real-time SARS-CoV-2 test and genome data to detailed epidemiological and clinical data on hospitalised cases. SGTF=S gene target failure.

From March 31, 2020, to Dec 6, 2021, there were 1734 hospitalised patients with COVID-19 for whom variant information was available, either by genome sequencing (non-variant, alpha, beta, or delta) or TaqPath PCR (SGTF as a proxy for the omicron variant between Oct 1 and Dec 6, 2021). Of these 1734, 792 (45·7%) were infected with the delta variant from April 1, 2021, (week 13) to Nov 9, 2021, (week 45), and 212 (12·2%) had SGTF infections from Oct 1, 2021, (week 39) to Dec 6, 2021 (week 49; figure 2).

Among 1142 hospitalised individuals, 1037 (90·8%) had accumulated an in-hospital outcome by Dec 21, 2021. After controlling for factors associated with severe disease, individuals with SGTF infections diagnosed between Oct 1 and Nov 30, 2021, had a significantly lower odds of severe disease than did those with delta variant infections diagnosed between April 1 and Nov 9, 2021, (aOR 0·3, 95% CI 0·2–0·5; table 4). In addition to variation by geographical province, older age (40–59 years and ≥60 years *vs* 19–24 years) and having a comorbid condition were associated with an increased odds of severe disease (table 4). Individuals aged 13–18 years (*vs* 19–24 years) and women (*vs* men) had lower odds of severe disease (table 4).

**Table 4:** Multivariable logistic regression analysis evaluating the association between SGTF infection during Oct 1–Nov 30, 2021, compared with delta variant infection during April 1–Nov 9, 2021, and severe disease among hospitalised individuals with a known outcome, South Africa* (n=1036)

	**Severe disease**†	**Adjusted odds ratio (95% CI)**	**p value**
**SARS-CoV-2 variant (n=1037)**			
SGTF	57/244 (23·4%)	0·3 (0·2–0·5)	<0·001
Delta (B.1.617.2)	496/793 (62·5%)	1 (ref)	..
**Age group (n=1037)**			
<5 years	7/41 (17·1%)	0·5 (0·2–1·6)	0·23
5–12 years	4/16 (25·0%)	0·7 (0·2–2·9)	0·61
13–18 years	3/22 (13·6%)	0·2 (0·0–0·8)	0·023
19–24 years	15/42 (35·7%)	1 (ref)	..
25–39 years	71/243 (29·2%)	0·8 (0·4–1·9)	0·66
40–59 years	189/309 (61·2%)	2·2 (1·0–5·0)	0·048
≥60 years	264/364 (72·5%)	3·8 (1·7–8·6)	0·001
**Sex (n=1036)**			
Male	248/425 (58·4%)	1 (ref)	..
Female	305/611 (49·9%)	0·7 (0·5–1·0)	0·032
**Province (n=1037)**			
Eastern Cape	42/47 (89·4%)	3·5 (1·2–10·3)	0·023
Free State	2/4 (50·0%)	1·1 (0·1–24·3)	0·94
Gauteng	210/432 (48·6%)	1·1 (0·7–1·9)	0·61
KwaZulu-Natal	28/48 (58·3%)	2·1 (0·9–4·7)	0·089
Limpopo	78/103 (75·7%)	1·9 (1·0–3·7)	0·050
Mpumalanga	44/60 (73·3%)	1·8 (0·8–4·0)	0·13
North West	87/148 (58·8%)	1 (ref)	..
Northern Cape	23/26 (88·5%)	5·9 (1·4–24·3)	0·014
Western Cape	39/169 (23·1%)	0·1 (0·1–0·2)	<0·001
**Comorbidity**‡**(n=1037)**			
Absent	270/658 (41·0%)	1 (ref)	..
Present	283/379 (74·7%)	2·8 (2·0–4·0)	<0·001
**Health-care sector (n=1037)**			
Public	472/787 (60·0%)	1 (ref)	..
Private	81/250 (32·4%)	0·7 (0·4–1·3)	0·28
**Days between diagnosis and admission (n=1037)**			
1–7 days before diagnosis	154/267 (57·7%)	1 (ref)	..
0–6 days after diagnosis	351/686 (51·2%)	0·7 (0·5–1·0)	0·061
7–21 days after diagnosis	48/84 (57·1%)	0·9 (0·5–1·8)	0·85
**Reinfection**§**(n=1037)**			
No	541/1000 (54·1%)	1 (ref)	..
Yes	12/37 (32·4%)	1·1 (0·5–2·7)	0·80
**SARS-CoV-2 vaccination**¶**(n=1037)**			
No	141/207 (68·1%)	1 (ref)	..
Yes	21/38 (55·3%)	0·6 (0·2–1·5)	0·25
Unknown	391/792 (49·4%)	1·1 (0·7–1·7)	0·79

Data are n/N (%), unless otherwise specified. SGTF=S gene target failure.

* Patients with COVID-19 followed up for in-hospital outcome until Dec 21, 2021.

† Severe disease was defined as a hospitalised patient meeting at least one of the following criteria: admitted to an intensive care unit; received oxygen treatment; was ventilated; received extracorporeal membrane oxygenation; had acute respiratory distress syndrome; or had died.

‡ Comorbidity was defined as the presence of at least one of the following conditions: hypertension, diabetes, chronic cardiac disease, chronic kidney disease, asthma, chronic obstructive pulmonary disease, malignancy, HIV, and active or previous tuberculosis.

§ Reinfection was defined by an individual having at least one positive SARS-CoV-2 test more than 90 days before the current positive SARS-CoV-2 test.

¶ Vaccination was defined as having at least one dose of a SARS-CoV-2 vaccine (Ad.26.COV2.S [Johnson & Johnson] or BNT162b2 [Pfizer–BioNTech]).

## Discussion

In November, 2021, the omicron SARS-CoV-2 variant emerged in South Africa, concurrent with a rapid increase in the number of reported COVID-19 cases,^2^ leading to the fourth epidemic wave. Although numerous studies are underway to rapidly assess phenotypic behaviour and immune escape, it is also crucial to understand the clinical severity of infections with the omicron variant. We evaluated the severity of SGTF infections (a proxy for the omicron variant) by comparing them to non-SGTF infections diagnosed during the same period and to infections with the delta variant, which dominated the third wave in South Africa. Individuals with SGTF versus non-SGTF infections had an 80% lower odds of being admitted to hospital, but we were not able to make any firm conclusions on the risk of severe disease among hospitalised people, possibly due to the low numbers of individuals included in this analysis. When compared with delta variant infections, SGTF infections were associated with a 70% lower odds of severe disease. In addition, we found that the mean PCR cycle threshold was lower during the early wave of omicron than during the early wave of delta, which might reflect higher viral loads in individuals infected with the omicron variant versus the delta variant.

Data from the DATCOV surveillance programme have shown a higher proportion of admissions among people younger than 20 years during the early fourth wave compared with the third wave.^11^ In our study, we also found that SGTF-infected individuals were younger than those with non-SGTF or delta infections; this effect was not restricted to children as there were also more SGTF infections (*vs* non-SGTF or delta) in younger adult age groups (eg, 19–24 years) than in the older adult age group (≥60 years). This finding could be related to increased immunity in older adults as a result of higher rates of previous infection^12^ or vaccination.^13^

Our findings correlate with hospitalisation data showing that, among 1061 patients in South Africa with COVID-19 and a known hospital outcome, 324 (30·5%) had severe disease during the early fourth wave, compared with 943 (67·0%) of 1408 patients during the early third wave.^11^ By November, 2021, a high proportion of the South African population had some level of SARS-CoV-2 immunity as a result of previous natural infection, vaccination, or both. After the third SARS-CoV-2 wave, 60–70% of individuals in South Africa had evidence of previous SARS-CoV-2 infection.^14^ By Dec 9, 2021, 58% of South African individuals aged 60 years or older, 55% aged 50–59 years, 43% aged 35–49 years, and 24% aged 18–34 years were fully vaccinated against SARS-CoV-2 (one dose of Ad.26.COV2.S [Johnson & Johnson] or two doses of BNT162b2 [Pfizer–BioNTech]).^13^ It has been suggested that the omicron variant has substantially increased immune escape capabilities compared with other variants.^15^ This finding, together with the high levels of pre-existing immunity in South Africa at the time of emergence of the omicron variant, means that a high proportion of omicron infections are likely to be reinfections and that this proportion might be greater for omicron than for contemporaneous infections with other variants. In South Africa, during the first and second waves of COVID-19, fewer than one in ten cases of SARS-CoV-2 were diagnosed.^16^ We accounted for individuals with diagnosed and previously reported infections in our analysis, but, in many cases, it is probable that previous infection could have been undiagnosed. If reinfections are less severe than primary infections, then this fact could, in part, account for the reduced disease severity observed in individuals infected by the omicron variant.^17^ It is also possible that the omicron variant has reduced intrinsic virulence compared with other variants.^18^, ^19^ With the available data, it is not possible to disentangle the relative contributions of high levels of population immunity versus lower intrinsic virulence to the observed lower disease severity of the omicron versus delta variant. Incomplete vaccination data, and the fact that many previous infections were probably not detected, resulted in incomplete adjustment for the effect of previous immunity in our analyses.

This study has several limitations. First, SGTF infections were only identifiable when the TaqPath COVID-19 PCR test was used and for specimens with high viral loads (cycle threshold ≤30); therefore, the number of SGTF infections is underestimated and biased towards geographical regions where the TaqPath assay was more commonly used. Second, SGTF was used as a proxy for omicron variant detection. SGTF might also identify the alpha variant and occur sporadically in other variants, and so some infections might have been misclassified as omicron. However, we only used SGTF as a proxy for omicron after Oct 1, 2021, and genome data generated by the NGS-SA have not identified the alpha variant in South Africa since August, 2021, and, at its peak, the alpha variant was only detected in 58 (6%) of 965 samples in May, 2021.^20^ Omicron has been classified into three sub-lineages, one (BA.2) of which does not contain His69_Val70del and is therefore not identifiable by SGTF. Genome data from November, 2021, in South Africa showed that, of 881 omicron sequences, 872 (99%) were BA.1, one (<1%) was BA.2, and eight (1%) were BA.3.^20^ Although genome data for non-SGTF infections were not available, of samples sequenced in South Africa, 617 (84·2%) of 733 were delta and one (0·1%) of 733 was omicron in October, 2021, and 148 (13·7%) of 1082 were delta and 881 (81·4%) of 1082 were omicron in November, 2021, suggesting that the majority of non-SGTF infections were delta.^20^ Third, our analysis was done during the early fourth wave, in which numbers of people with COVID-19 were small, patients with milder symptoms were more likely to be admitted to hospital (compared with peak wave periods), and there could have been a lag in hospitalisations and severe disease outcomes caused by the omicron variant. We accounted for this limitation by only including hospitalised patients with known outcomes, censoring cases to ensure at least 3 weeks of follow-up, and adjusting for the time interval between diagnosis and hospitalisation in the severity models. The inclusion of individuals only with accumulated in-hospital outcomes could have biased individuals with SGTF infections towards shorter hospital stays; hence, this result should be interpreted with caution. Fourth, we compared individuals with COVID-19 during the full delta wave to those with COVID-19 during the ascending phase of the omicron wave, which could have biased comparisons if case characteristics differ between ascending and descending wave phases or if the threshold for hospitalisation changed in the different time periods. Data from the DATCOV programme suggest that the proportion of patients with severe COVID-19 does not vary substantially through the different wave phases.^11^ Finally, we only had information on vaccination status for hospitalised individuals with COVID-19, which was also based on self-reporting.

Despite the omicron variant being detected only recently, there has been a rapid increase in the prevalence of SGTF infections across all South African provinces during November, 2021, with almost complete replacement of the delta variant in a period of 4 weeks. Our early analyses indicate a significantly reduced risk of hospital admission among SGTF-infected individuals compared with non-SGTF-infected individuals diagnosed between Oct 1 and Nov 30, 2021, and a reduced risk of severe disease among SGTF-infected individuals compared with delta variant-infected individuals. Immunity (due to previous infection, vaccination, or both) in individuals infected with the omicron variant might, in part, account for this reduced severity. Potential reduced intrinsic virulence with regards to the omicron variant might also contribute to observed reductions in COVID-19 severity. Future studies that link data on vaccination status from vaccine registries could provide information on the attenuation of disease severity in breakthrough cases of COVID-19 following vaccination. These data are early and findings could change with additional follow-up time for the more recently diagnosed SGTF-infected individuals.

## Data sharing

The deidentified individual participant dataset used in this Article will be made available with publication upon reasonable request. Proposals should be directed to cherylc@nicd.ac.za. Data will be provided upon provision of protocol and ethics approval for the proposed study and a signed data sharing agreement.

## Declaration of interests

CC has received grant support from the South African Medical Research Council, the UK Foreign, Commonwealth and Development Office, the Wellcome Trust, the US Centers for Disease Control and Prevention, and Sanofi Pasteur. NW and MdP have received grant support from Sanofi Pasteur and the Bill & Melinda Gates Foundation. AvG has received grant support from Sanofi Pasteur, the US Centers for Disease Control and Prevention, the South African Medical Research Council, the Bill & Melinda Gates Foundation, WHO, the Fleming Fund, and the Wellcome Trust. RW declares personal shareholding in Adcock Ingram Holdings, Dischem Pharmacies, Discovery, Netcare, and Aspen Pharmacare Holdings. WS has received grant support from the South African Medical Research Council, with funds received from the Department of Science and Innovation, and the Bill & Melinda Gates Foundation. All other authors declare no competing interests.
