# Supplementary material for: Early assessment of the clinical severity of the SARS-CoV-2 omicron variant in South Africa: a data linkage study
Source: Lancet. 2022 Jan 29;399(10323):437–46. doi: 10.1016/S0140-6736(22)00017-4 (PMC8769664; doi:10.1016/S0140-6736(22)00017-4)
Supplement: Supplementary appendix [file mmc1.pdf]

THE LANCET

Supplementary appendix

This appendix formed part of the original submission and has been peer reviewed. We post it as supplied by the authors.

Supplement to: Wolter N, Jassat W, Walaza S, et al. Early assessment of the clinical severity of the SARS-CoV-2 omicron variant in South Africa: a data linkage study. *Lancet* 2022; published online Jan 19. https://doi.org/10.1016/S0140-6736(22)00017-4.

Supplement

Supplementary Table 1. Multivariable logistic regression analysis evaluating factors associated with S gene target failure (SGTF) infection compared to non-SGTF infection among hospitalised individuals, 1 October – 30 November 2021[a] (N=302)

		Non-SGTF[b] n (%)	SGTF[b] n/N (%)	Odds ratio (95% CI)	Adjusted odds ratio (95% CI)	P-value
**Age group (years)**		N=113	N=204			
	<5	7 (6)	20 (10)	3.5 (1.6-9.7)	1.0 (0.3-3.4)	0.962
	5-12	2 (2)	6 (3)	3.7 (0.7-20.0)	1.9 (0.3-11.9)	0.498
	13-18	2 (2)	6 (3)	3.7 (0.7-20.0)	1.7 (0.3-11.0)	0.559
	19-24	3 (3)	20 (10)	8.3 (2.2-31.0)	7.5 (1.5-38.1)	**0.016**
	25-39	35 (31)	101 (50)	3.6 (1.9-6.9)	2.0 (0.9-4.7)	0.096
	40-59	33 (29)	26 (13)	1.0 (0.5-2.0)	0.8 (0.3-1.8)	0.535
	≥60	31 (27)	25 (13)	Ref	Ref	-
**Sex**		N=113	N=202			
	Male	47 (42)	73 (36)	Ref	Ref	-
	Female	66 (58)	129 (64)	1.3 (0.8-2.0)	1.2 (0.7-2.2)	0.466
**Province**		N=107	N=201			
	Eastern Cape	0 (0)	0 (0)	-	-	-
	Free State	5 (5)	3 (1)	3.0 (0.4-24.2)	1.4 (0.1-15.8)	0.805
	Gauteng	64 (60)	166 (83)	13.0 (2.8-60.8)	4.3 (0.8-22.5)	0.086
	KwaZulu-Natal	21 (20)	17 (8)	4.0 (0.8-21.0)	1.2 (0.2-7.5)	0.833
	Limpopo	0 (0)	5 (2)	-	-	-
	Mpumalanga	1 (1)	3 (1)	15.0 (1.0-228.9)	4.3 (0.2-85.7)	0.342
	North West	3 (3)	4 (2)	6.7 (0.8-56.2)	1.6 (0.2-15.8)	0.691
	Northern Cape	3 (3)	1 (1)	1.7 (0.1-25.4)	0.9 (0.0-18.3)	0.962
						-
	Western Cape	10 (9)	2 (1)	Ref	Ref	
**Healthcare sector**		N=113	N=204			

	Public	68 (60)	62 (30)	Ref	Ref	-
	Private	45 (40)	142 (70)	3.5 (2.1-5.6)	3.1 (1.6-5.7)	**<0.001**
**Days between diagnosis and admission**		N=113	N=204			
	1-7 days before diagnosis	17 (15)	21 (10)	Ref	Ref	**-**
	0-6 days after diagnosis	93 (82)	182 (89)	1.6 (0.8-3.1)	0.9 (0.4-2.3)	0.906
	7-21 days after diagnosis	3 (3)	1 (0)	0.3 (0.0-2.8)	0.5 (0.0-7.1)	0.593
**Reinfection**[c]		N=113	N=204			
	No	107 (95)	183 (90)	Ref	Ref	-
	Yes	6 (5)	21 (10)	2.0 (0.8-5.2)	2.2 (0.6-7.5)	0.218
**Co-morbidity**[d]		N=113	N=204			
	Absent	77 (68)	178 (87)	Ref	Ref	-
	Present	36 (32)	26 (13)	0.3 (0.2-0.6)	0.6 (0.3-1.3)	0.197
**SARS-CoV-2 vaccination**[e]		N=113	N=204			
	No	18 (16)	24 (12)	Ref	Ref	-
	Yes	5 (4)	4 (2)	0.6 (0.1-2.6)	0.3 (0.1-2.2)	0.254
	Unknown	90 (80)	176 (86)	1.5 (0.8-2.8)	0.9 (0.4-2.3)	0.857
**Severe disease**[f]		N=113	N=204			
	No	68 (60)	162 (79)	Ref	Ref	-
	Yes	45 (40)	42 (21)	0.4 (0.2-0.7)	0.7 (0.3-1.5)	0.353

[a] Cases followed-up for in-hospital outcome until 21 December 2021

[b] S gene target failure (proxy for Omicron variant)

[c] Re-infection was defined as an individual with at least one positive SARS-CoV-2 test >90 days prior to the current episode

[d] Co-morbidity defined as ≥1 of the following conditions: hypertension, diabetes, chronic cardiac disease, chronic kidney disease, asthma, chronic obstructive pulmonary disease (COPD), malignancy, HIV, and active or past tuberculosis

[e] Vaccination defined as ≥1 dose of SARS-CoV-2 vaccine (Johnson & Johnson / Pfizer-BioNTech)

[f] Severe disease defined as a hospitalized patient meeting at least one of the following criteria: admitted to ICU, received oxygen treatment, ventilated, received extracorporeal membrane oxygenation (ECMO), experienced acute respiratory distress syndrome (ARDS) and/or died

Supplementary Table 2. Multivariable logistic regression analysis evaluating factors associated with S gene target failure (SGTF) infection during 1 October – 30 November 2021, compared to Delta variant infection during April – November 2021, among hospitalized individuals with known outcome[a], South Africa (N=989)

		Delta n (%)	SGTF[b] n/N (%)	Odds ratio (95% CI)	Adjusted odds ratio (95% CI)	P-value
**Age group (years)**		N=793	N=244			
	<5	21 (3)	20 (8)	11.4 (5.5-23.6)	7.6 (1.9-30.7)	**0.005**
	5-12	9 (1)	7 (3)	9.3 (3.2-26.9)	13.4 (1.6-111.8)	**0.016**
	13-18	18 (2)	4 (2)	2.7 (0.8-8.4)	5.8 (0.7-49.2)	0.107
	19-24	21 (3)	21 (9)	12.0 (5.9-24.6)	15.0 (3.8-58.9)	**<0.001**
	25-39	123 (16)	120 (49)	11.7 (7.4-18.6)	9.7 (4.3-21.7)	**<0.001**
	40-59	265 (33)	44 (18)	2.0 (1.2-3.3)	1.4 (0.6-3.0)	0.404
	≥60	336 (42)	28 (11)	Ref	Ref	-
**Sex**		N=792	N=244			
	Male	330 (42)	95 (39)	Ref	Ref	-
	Female	462 (58)	149 (61)	1.1 (0.8-1.5)	0.7 (0.4-1.2)	0.240
**Province**		N=793	N=244			
	Eastern Cape	47 (6)	0 (0)	-	-	-
	Free State	2 (0)	2 (1)	83.5 (7.5-925.3)	129.2 (4.0-4183.8)	**0.006**
	Gauteng	232 (29)	200 (82)	72.0 (17.6-293.9)	25.3 (5.5-117.0)	**<0.001**
	KwaZulu-Natal	26 (3)	22 (9)	70.6 (15.7-318.3)	34.5 (55-216.7)	**<0.001**
	Limpopo	99 (12)	4 (2)	3.4 (0.6-18.8)	1.8 (0.2-14.8)	0.564
	Mpumalanga	55 (7)	5 (2)	7.6 (1.4-40.2)	0.7 (0.1-4.9)	0.734

	North West	140 (18)	8 (3)	4.8 (1.0-22.8)	1.5 (0.2-9.0)	0.651
	Northern Cape	25 (3)	1 (0)	3.3 (0.3-38.2)	7.6 (0.5-105.7)	0.131
	Western Cape	167 (21)	2 (1)	Ref	Ref	-
**Healthcare sector**		N=793	N=244			
	Public	737 (93)	50 (20)	Ref	Ref	-
	Private	56 (7)	194 (80)	51.1 (33.8-77.2)	27.6 (14.6-52.3)	**<0.001**
**Days between diagnosis and admission**		N=793	N=244			
	1-7 days before diagnosis	249 (31)	18 (7)	Ref	Ref	**-**
	0-6 days after diagnosis	487 (61)	199 (82)	5.7 (3.4-9.4)	2.1 (0.9-4.9)	0.084
	7-21 days after diagnosis	57 (7)	27 (11)	6.6 (3.4-12.7)	1.5 (0.5-4.9)	0.479
**Reinfection[c]**		N=793	N=244			
	No	785 (99)	215 (88)	Ref	Ref	-
	Yes	8 (1)	29 (12)	13.2 (6.0-29.4)	22.7 (4.3-120.6)	**<0.001**
**Co-morbidity[d]**		N=793	N=244			
	Absent	449 (57)	209 (86)	Ref	Ref	-
	Present	344 (43)	35 (14)	0.2 (0.1-0.3)	1.0 (0.5-1.9)	0.956
**SARS-CoV-2 vaccination[e]**		N=793	N=244			
	No	185 (23)	22 (9)	Ref	Ref	-
	Yes	30 (4)	8 (3)	2.2 (0.9-5.5)	2.3 (0.4-12.1)	0.341
	Unknown	578 (73)	214 (88)	3.1 (1.9-5.0)	1.8 (0.8-4.0)	0.150
**Severe disease[f]**		N=793	N=244			
	No	297 (37)	187 (77)	Ref	Ref	-

Yes	496 (63)	57 (23)	0.2 (0.1-0.3)	0.3 (0.1-0.5)	**<0.001**

[a] Cases followed-up for in-hospital outcome until 21 December 2021

[b] S gene target failure (proxy for Omicron variant)

[c] Re-infection was defined as an individual with at least one positive SARS-CoV-2 test >90 days prior to the current episode

[d] Co-morbidity defined as ≥1 of the following conditions: hypertension, diabetes, chronic cardiac disease, chronic kidney disease, asthma, chronic obstructive pulmonary disease (COPD), malignancy, HIV, and active or past tuberculosis

[e] Vaccination defined as ≥1 dose of SARS-CoV-2 vaccine (Johnson & Johnson / Pfizer-BioNTech)

[f] Severe disease defined as a hospitalized patient meeting at least one of the following criteria: admitted to ICU, received oxygen treatment, ventilated, received extracorporeal membrane oxygenation (ECMO), experienced acute respiratory distress syndrome (ARDS) and/or died

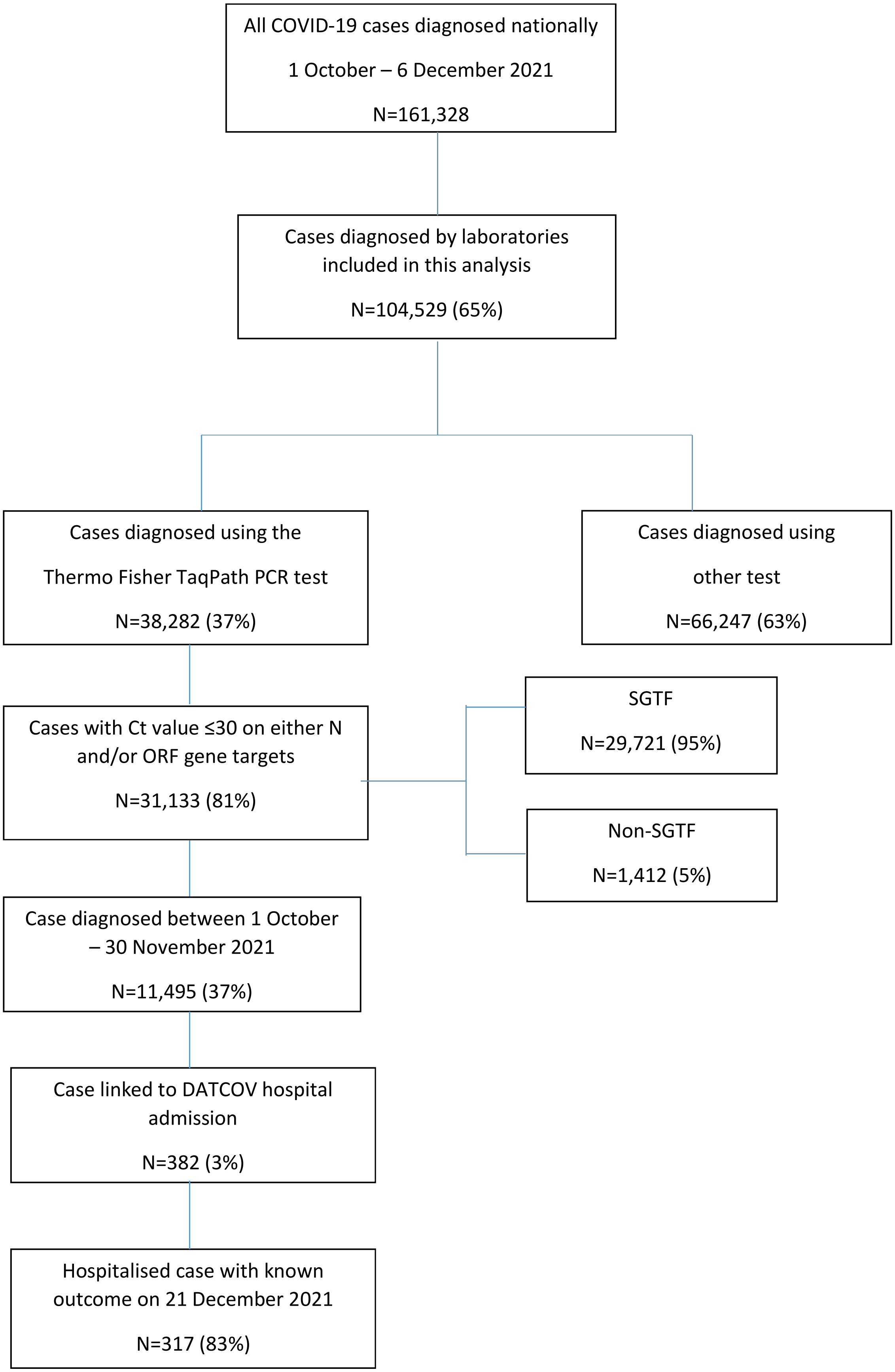
All COVID-19 cases diagnosed nationally
1 October – 6 December 2021
N=161,328
Cases diagnosed by laboratories included in this analysis
N=104,529 (65%)
Cases diagnosed using the Thermo Fisher TaqPath PCR test
N=38,282 (37%)
Cases diagnosed using other test
N=66,247 (63%)
Cases with Ct value ≤30 on either N and/or ORF gene targets
N=31,133 (81%)
SGTF
N=29,721 (95%)
Non-SGTF
N=1,412 (5%)
Case diagnosed between 1 October – 30 November 2021
N=11,495 (37%)
Case linked to DATCOV hospital admission
N=382 (3%)
Hospitalised case with known outcome on 21 December 2021
N=317 (83%)

Supplementary Figure 1. Flowchart of COVID-19 cases diagnosed in South Africa between 1 October – 6 December 2021 and linkage to test and hospitalisation data

PCR – polymerase chain reaction, Ct – cycle threshold, SGTF - S gene target failure

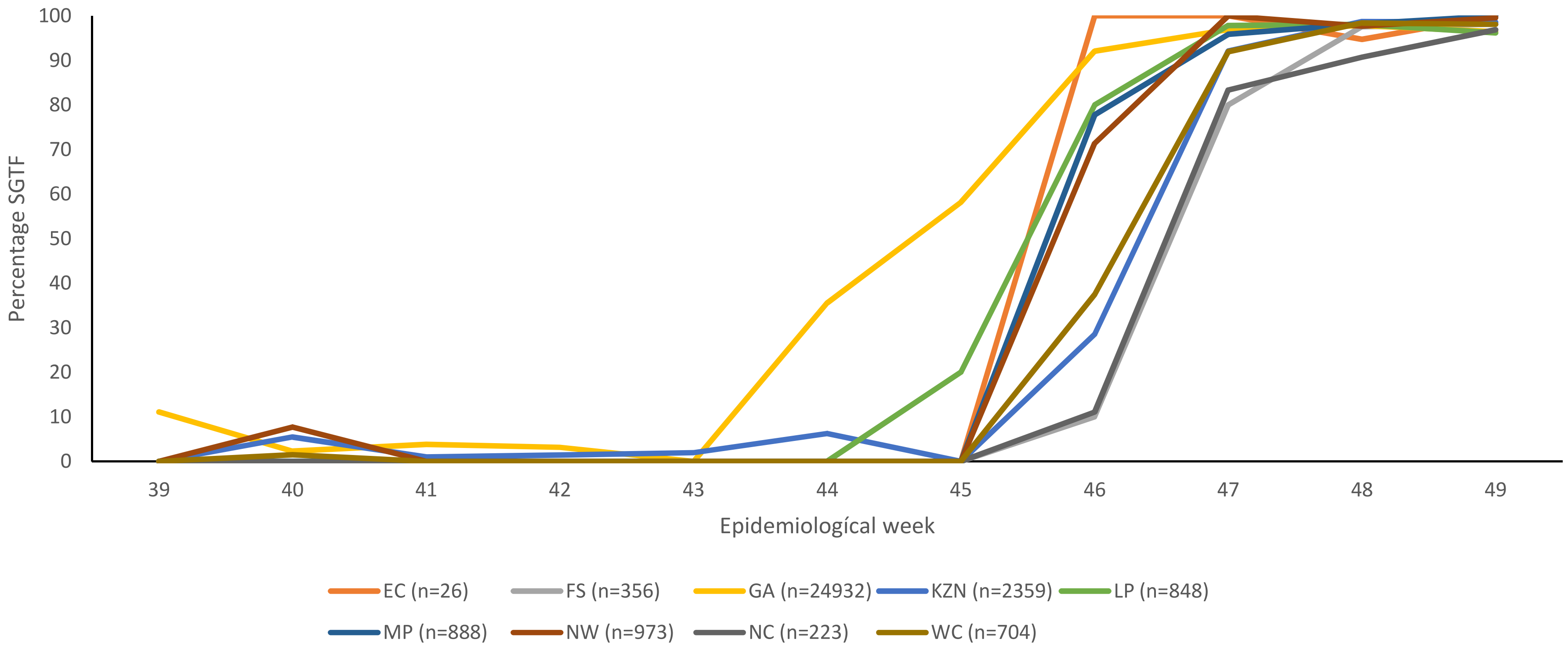

Supplementary Figure 2. Percentage of S gene target failure (SGTF) infections among tests performed on the TaqPath assay by epidemiological week and province, DATCOV-Gen, 1 October – 6 December 2021. GA, Gauteng; NW, North West; KZN, KwaZulu-Natal; WC, Western Cape; LP, Limpopo; EC, Eastern Cape; MP, Mpumalanga; NC, Northern Cape; FS, Free State
